# Brazzein and Monellin: Chemical Analysis, Food Industry Applications, Safety and Quality Control, Nutritional Profile and Health Impacts

**DOI:** 10.3390/foods12101943

**Published:** 2023-05-10

**Authors:** Ariana Saraiva, Conrado Carrascosa, Fernando Ramos, Dele Raheem, Sónia Pedreiro, Angelo Vega, António Raposo

**Affiliations:** 1 Department of Animal Pathology and Production, Bromatology and Food Technology, Faculty of Veterinary, Universidad de Las Palmas de Gran Canaria, Trasmontaña s/n, 35413 Arucas, Spain; 2 Faculty of Pharmacy, University of Coimbra, Azinhaga de Santa Comba, 3000-548 Coimbra, Portugal; 3 Associated Laboratory for Green Chemistry (LAQV) of the Network of Chemistry and Technology (REQUIMTE), Rua D. Manuel II, Apartado 55142, 4051-401 Porto, Portugal; 4 Arctic Centre, University of Lapland, 96101 Rovaniemi, Finland; 5 CBIOS (Research Center for Biosciences and Health Technologies), Universidade Lusófona de Humanidades e Tecnologias, Campo Grande 376, 1749-024 Lisboa, Portugal

**Keywords:** alternative sweeteners, food industry, nutrition, brazzein, monellin, chemical analysis, health impacts

## Abstract

Recently, customers have been keener to buy products manufactured using all-natural ingredients with positive health properties, but without losing flavor. In this regard, the objective of the current study is to review the consumption of brazzein and monellin, their nutritional profiles and health effects, and their potential applications in the food industry. This poses challenges with sustainability and important quality and safety indicators, as well as the chemical processes used to determine them. To better understand the utilization of brazzein and monellin, the chemical analysis of these two natural sweet proteins was also reviewed by placing particular emphasis on their extraction methods, purification and structural characterization. Protein engineering is considered a means to improve the thermal stability of brazzein and monellin to enhance their application in food processing, especially where high temperatures are applied. When the quality and safety of these sweet proteins are well-investigated and the approval from safety authorities is secured, the market for brazzein and monellin as food ingredient substitutes for free sugar will be guaranteed in the future. Ultimately, the review on these two natural peptide sweeteners increases the body of knowledge on alleviating problems of obesity, diabetes and other non-communicable diseases.

## 1. Introduction

One of the major worldwide dietary concerns is excessive intake of nutritive (caloric) sugars. According to a recent report, the average American consumes 17 teaspoons (tsp.) of added sugar daily. This is approximately twice the amount advised for males (9 tsp.) and females (6 tsp.) [1]. As this dietary practice poses several negative health effects, including a higher risk of diabetes, obesity and cardiovascular illnesses, as well as high blood pressure, efforts must be made worldwide to reduce sugar intake [2]. Currently, people in Canada consume 11–13% of their daily calorie intake from sugar [3], compared to children and adolescents in the USA, who consume 17% more sugar [4]. There is an ever-increasing need for non-nutritive (low-/zero-calorie) and safer sugar substitutes as a result of increased knowledge about sugar consumption behavior and accompanying health-related repercussions. There are several sweeteners on the market that satiate consumer appetites for sweetness, but each sweetener has specific applications and limitations [5,6].

In a variety of applications, artificial sweeteners (ATSs) have become more popular as sugar substitutes, but debate about their safety and long-term health implications is ongoing [2]. For instance, ATS usage impacts glucose homeostasis, changes the host microbiome, decreases satiety, increases calorie intake and causes weight gain [7]. Additionally, consuming the commonly used ATS aspartame is connected to several health-type side effects, including gastrointestinal problems, mood changes, dizziness and headaches [8]. ATSs have also been regarded as an environmental pollutant because they are used in food items and then reach the environment, where they may degrade or change into harmful compounds [9]. The production of natural sugar alternatives using natural resources has, therefore, gained new interest in meeting consumer dietary needs [10]. Brazzein, curculin, lysosyme, mabinlin, miraculin, monellin, pentadin and thaumatin are just a few of the proteins identified as having a sweet flavor to date [11,12,13,14,15,16,17]. Save the lysozyme protein, which is derived from egg whites, all these proteins were initially produced and extracted from tropical plants.

In light of the aforementioned criticisms, excess sugar intake is one of the major dietary issues in many parts of the world because it is linked with several health issues, including high blood pressure and a higher risk of diabetes, cardiovascular diseases and obesity. As breaking this eating habit is difficult, low- or sugar-free foods and beverages are essential and are made possible by high-value-added bio-ingredient sweeteners. The food business is implicated in using several potent sweeteners, most of which are synthesized instead of sugar (sucrose). Customers are more eager to purchase items made with all-natural components and beneficial health characteristics, but without sacrificing flavor. Natural sweeteners have been launched by the food industry as an option to address this trend and to offer customers potential health advantages. Many of the high-value elements and biomolecules found in nature, including proteins with a sweet taste, are yet unexplored. To demonstrate the safety of natural components employed as additives or food supplements, such as sweeteners, it is important to perform extensive and in-depth scientific research [18].

### 1.1. Brazzein

*Pentadiplandra brazzeana* Baillon (*P. brazzeana*) naturally grows in tropical African forests and is the source of brazzein [19]. The structure of the smallest sweet-tasting protein, brazzein, is made up of 54 amino acids [20]. Brazzein stability remains up to 80 °C [21], which is a significant characteristic for food processing. Brazzein sweetness potency is 750-fold higher than sucrose by weight at the threshold level [22]. 

Due to the original plant’s location and brazzein production being restricted, different methods are sought. The best possible natural method for producing brazzein is by bioconversion. *Escherichia coli* was used in the first brazzein biotransformation research, which was conducted in 2000 [23]. However, further brazzein biotransformation tests in *E. coli* have shown a lower sweetness level than the original plant can produce. Later, *Pichia* was used to manufacture sweet brazzein. In only 6 days, the brazzein secreted by *Pischia* cells was roughly 120 mg/L. However, in a short time, *Kluyveromyces lactis* generated roughly 104 mg/L of brazzein in cultured medium, and the sensory properties of the recombinant brazzein were comparable to those of the original plant product [19]. *Bacillus licheniformis* has been recently used to extract brazzein because of its quick growth, high secretion and inexpensive cost [13]. After 36 h, 57 mg/L of brazzein was created as a result of Brazzein genes being expressed. The sweetness properties of Bbrazzein and Ebrazzein were 266-fold and 400-fold greater than those of sucrose, respectively.

The most frequently used mediums for brazzein plant biotransformation research purposes are lettuce, maize and rice [24,25]. Furthermore, brazzein is developed to yield 400 µg/g in maize seeds, and maize brazzein enables industrial manufacturing that can address problems with the original brazzein’s long-term viability [19]. As brazzein’s sweet flavor develops more gradually than that of sucrose, it can be used instead of sugar in innovative food applications.

### 1.2. Monellin

Monellin contains 50 amino acids in one chain and 44 in another one, which are bound together by polypeptide bonds [26]. The *Dioscoreophyllum cumminsii* Diels plant grows naturally in African forests and produces monellin, a protein with a sweet flavor [19]. On a weight basis, monellin has a sweetness that is 4000-fold greater than sucrose [27].

Except in natural environments in attempts to acquire stable monellin, *Dioscoreophyllum* cultivation trials have not been successful [28]. Thus, interest has been shown in biotransformation experiments, and a particular monellin form confers biotransformation flexibility. By way of example, monellin conversion by *E. coli* maintains a post-heating sweet flavor with greater pH stability than the original substance [26]. Additionally, 54 g of monellin, which has been purified, has been generated via monellin biotransformation with *Saccharomyces cerevisiae* [29].

Transgenic tomato and lettuce have been used in monellin plant experiments [30]. As a result, transgenic tomato has been exposed to ethylene to produce 23.9 µg/g of monellin with good thermal stability and enhanced sweetness [31].

To identify long-term solutions for the component’s numerous food production applications, monellin biotransformation studies will continue. As monellin’s glycemic index is low, it can be added to diabetics’ diets [32]. In addition, no negative monellin impacts for food applications have yet been observed [33], and the compound can soon be used in a variety of food processing processes.

These findings guide the present review’s objective to examine the health effects and nutritional profile of brazzein and monellin intake, sustainability concerns, some potential food industry applications, key safety and quality indicators, and the chemical analyses involved in their determination.

## 2. Chemical Analysis

### 2.1. Brazzein

Brazzein was reported for the first time in 1994 by Ming and Hellekant as a new sweet and thermostable protein that can be isolated from the fruit of *P. brazzeana* [34]. The thermostability of the brazzein solution was evaluated by incubation at 98 °C for 2 h and at 80 °C for 4.5 h. The results show that brazzein does not lose sweetness, which is an indicator of protein stability at high temperature [34]. In addition to its sweetness and thermostability, brazzein exhibits high water solubility (more than 7.7 mM) and an isoelectric point of 5.4 [34,35]. 

Brazzein can be extracted from *P. brazzeana* using buffer solutions, such as phosphate buffer, and can then be precipitated with ammonium sulfate [34,36]. However, growing *P. brazzeana* is difficult, and brazzein production and extraction have low yields and are expensive. Several authors have resorted to the technological production of this protein using bacteria [37,38,39,40,41,42], yeasts [43,44], transgenic plants [35,36] and animals [35,45]. Once the protein is extracted or expressed, it is isolated, purified and characterized. Table 1 contains the main brazzein isolation, purification and characterization methods.

#### 2.1.1. Isolation and Purification

According to Table 1, isolation of brazzein is commonly achieved by precipitation with 30–85% ammonium sulfate [34,36,46,47,48,49]. Brazzein is purified mainly by ion exchange chromatography using carboxymethyl Sepharose or diethylaminoethyl (DEAE) columns. Other techniques are also applied, such as metal affinity chromatography using nickel or cobalt columns, and RT-HPLC. 

#### 2.1.2. Structural Characterization 

Ming and Hellekant were the first to characterize brazzein by SDS-PAGE, ESI-MS, Edman degradation and RT-HPLC [34]. Brazzein is a single-chain polypeptide whose tyrosine is the C-terminal amino acid. The molar masses obtained by SDS-PAGE and ESI-MS were 6.5 KDa and 6.473 KDa, respectively. Of 54 residues, 8 are cysteines. By means of a sequence computational analysis, curculin is the most homologous to brazzein by sharing 20% identity in a 50 amino acid overlap [34]. Two naturally occurring forms of brazzein have been reported. The major form, which represents 80% of brazzein in seed, is composed of 54 amino acid residues and has a pyroglutamic acid residue at the N-terminal. The minor form (20%), composed of 53 amino acid residues, lacks the N-terminal pyroglutamic acid residue [35]. Of these two forms, the minor one is reported as the sweeter [35]. All the amino acids in the brazzein structure are L-amino acids [50]. D-brazzein can be synthesized and is devoid of sweetness and taste [50].

As previously mentioned, the sweetness and thermostability characteristics of brazzein are consequences of the folding of primary and secondary structures. Thus, its structure is three-dimensional [34,35,47,51]. In this way, Caldwell et al. [47] employed 1H NMR spectroscopy and reported the three-dimensional brazzein structure for the first time [47]. Brazzein folding occurs due to a “cysteine-stabilized apha-beta motif” (CSαβ), which is responsible for the tabilization of the only α-helix with the nearest β-strand through two disulfide bonds [47]. Four disulfide bridges contribute to the folding and sweetness of brazzein [39,47]. In 1999, Gao et al. [49] studied the secondary brazzein structure at pH 3.5 by 1H NMR and published similar results. The secondary brazzein structure is composed of one α-helix and two antiparallel β-sheets. However, the authors also found a 310 α-helix and a third strand close to the N-terminal amino acid [49]. Nagata et al. studied the secondary brazzein structure by X-ray Crystallography at pH 4. They reported a 310 helix in the first loop between the first β-strand and the α-helix [48]. However, this 310 helix was found only at low pH and did not significantly influence the sweet taste of brazzein [48]. Over the years, both the structure and conformation of brazzein have been studied by many methods, namely RP-HPLC [39], NMR spectroscopy [38,39,47,49,52], X-ray Crystallography [4] and Circular Dichroism spectroscopy [36,40,53].

Table 1 includes information about the isolation, purification and characterization methods employed with brazzein and its mutations obtained by either *P. brazzeana* extraction or biotechnological processes. This section intends to discuss the purification and characterization methods of brazzein, thus the expression methods of recombinant brazzein are not discussed. According to Table 1, Edman degradation is the most widespread method used to determine the primary brazzein structure, and its secondary structure is determined mainly by Circular Dichroism, NMR and X-ray Crystallography. The tertiary structure is achieved mainly by Circular Dichroism, followed by fluorescence spectroscopy. The molar mass is determined mostly by SDS-PAGE and ESI-MS.

### 2.2. Monellin

Monellin is a sweet protein isolated from the fruit of *Dioscoreophyllum cumminsii* Diels [54]. Morris et al. identified monellin as a carbohydrate-free protein [54]. This protein has a molar mass of 10.7 KDa and an isoelectric point of 9.26 [55,56]. Unlike brazzein, monellin is not thermostable, and its sweetness is lost above 50 °C and at low pH values [55,56]. However, other authors have reported a molar mass of 10.5 KDa and reported protein denaturation at temperatures above 70 °C and high pH values [57]. Monellin’s sweet taste is stated to exist from pH 2 to 9 [57]. In relation to its structure, monellin is a heterodimer composed of two non-covalently linked peptidic chains: chain A with 44 amino acid residues and chain B with 50 amino acid residues [49]. It has only one thiol group, which has been associated with monellin’s sweetness [58]. To increase its thermostability, a derivative of monellin (MNEI) has been expressed. MNEI is a single chain composed of two monellin chains, where a Gly-Phe dipeptide fuses the N-terminal and C-terminal [57]. This derivative has the identical conformation as the double chain monellin, is stabler and also has a sweet taste [57]. The mutants of MNEI have been expressed and studied [57]. However, this section mainly focuses on monellin’s structural characterization and, therefore, the thermostability and monellin approaches are not discussed in detail.

#### 2.2.1. Isolation and Purification

According to Table 2, the isolation of brazzein is commonly achieved by salt precipitation [54,58]. Ion exchange chromatography is the most used method to perform the purification of monellin and its derivatives. Monellin has a net positive charge of 3 at pH 5.5. In this way, the binding of monellin with the Sepharose column is weak and allows good purification [59]. 

#### 2.2.2. Structural Characterization 

The secondary monellin structure is composed of five β-strands (β1–β5) that form a 17-residue α-helix and an antiparallel β-sheet [56]. In MNEI proteins, the secondary structure, determined by X-ray Crystallography, shows that β-sheets are closed by three main loops, and end with four proline residues in which a 310-polyproline II helix forms (confirmed by Circular Dichroism) [50]. No reports are available on the tertiary structure. According to Table 2, the monellin structure is determined mainly by SDS-PAGE, NMR, X-ray Crystallography, Edman degradation and Circular Dichroism.

## 3. Applications in the Food Industry and Sustainability Issues

**Figure 1:**
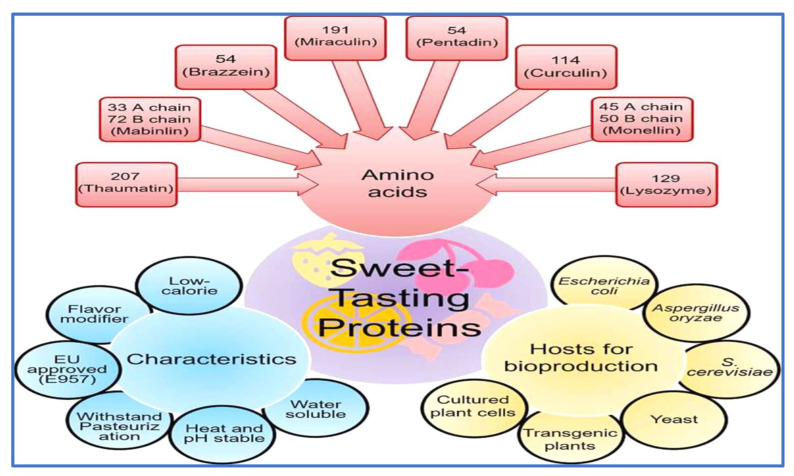
Amino acids, multifunctional characteristics and bioproduction hosts of sweet-tasting proteins [18].

There are certain characteristics that need to be considered when applying natural sweeteners of plant origin in food industries, which are reviewed here. Food industries rely on processing techniques to transform food ingredients, but these techniques have consequences on the final product. Overall, sweet-tasting proteins (including brazzein and monellin), for their successful utilization in the food industry, have to be contemplated for their low-calorie ability to withstand pasteurization, flavor-modifier effect, heat and pH stability and water solubility in colloidal solutions, as shown in Figure 1.

Commercial thaumatin exploitation as a sweetener and flavor enhancer has attracted more interest than other sweet-tasting proteins. Thaumatin can be prepared using the fruit of the tropical plant that produces it in economically convenient yields. However, neither brazzein nor monellin has been extracted from tropical plants in industrial quantities [16]. The thermal properties of brazzein and monellin have been a major barrier for their application in the food industry.

There is ongoing research on the key characteristics of each sweet protein, which are shown in Figure 1. These characteristics are important for the formulation of food products when food ingredients are considered. 

Sweet-tasting proteins’ low thermal stability has limited their food industry applications. As heat treatment is a commonplace procedure in the food industry, employing sweet-tasting proteins in this industry requires improving their protein stability. Protein engineering can help with this. To date, however, an efficient sweet-tasting protein engineering strategy is not widely accepted or considerably applied [67]. Studies reveal that some single-point mutations can improve these sweet-tasting proteins’ thermal stability, but these research works were conducted mainly according to random guess, which is time-consuming and very costly [66,68,69,70,71]. Lately, more attention has been paid to in silico approaches to apply target-oriented mutagenesis given its low cost and relatively good accuracy for high-throughput screening purposes. Mutating negatively charged residues to other non-negatively charged amino acids is an efficient way to improve the investigated sweet-tasting proteins’ thermal stability. Furthermore, some promising mutation sites have been identified to enhance thermal stability by mutagenesis [67]. The high cost of obtaining brazzein and monellin in their natural state has also led to genetic engineering to be applied to microbial hosts to mass produce them at a lower cost [18].

### 3.1. Brazzein

Despite being the most stable sweet-tasting protein, brazzein remains undenatured when heated to 80 °C for 4 h, and greater thermal stability is frequently necessary for it to be processed and applied to the food industry [45,72]. However, a recent high-intensity sweetener called Ultratia^TM^ brazzein (a small heat-stable protein that is from 500- to 2000-fold sweeter than regular sugar) was recently launched by the Swedish firm Sweegen in February 2022 (www.sweegen.com).

Protein engineering is an extremely useful tool for making these sweet-tasting proteins more appropriate for food industrial applications [73]. Single amino acid mutagenesis studies have improved sweet-tasting proteins’ thermal stability [68]. A similar research work by Ishikawa et al. observed that brazzein’s sweetness profile remained even after incubation at 353 K (79.85 °C) for 4 h, which was likely due to the compact structure that its four disulfide bridges afford [46]. Hence, either brazzein’s sweet taste or its potency is similar to that of sucrose, but with a slight aftertaste (Izawa et al., 2010 [55]), and it can thus readily replace sugar in food processes with novel food applications. Brazzein, as a natural peptide sweetener, can enhance flavor in beverages with citric acid and can be used to decrease the side taste of other sweeteners, such as stevioside, acesulfame-K and aspartame [74] (Hellekant and Danilova, 2005).

There are also sustainability aspects of using brazzein. For plant biotransformation studies with brazzein, the most widely applied media are lettuce, rice and maize [24]. Brazzein can be obtained by production in approximately 400 µg/g maize seeds, and maize brazzein enables industrial production to overcome issues related to the original brazzein’s sustainability [75].

There is a possibility of producing presweetened cereals with “no added sugar” by expressing the brazzein protein in maize seed embryos [76].

### 3.2. Monellin

Monellin is one of the most widely investigated sweet-tasting proteins whose thermal stability is greater when E23 is mutated to A, L, F, W or Q. Lesser thermal stability has been observed when Y65 is mutated to R [28,45]. These findings clearly suggest that single amino acid mutagenesis can help us to prepare sweet-tasting proteins that better resist thermal denaturation. Owing to the high cost of monellin, its current commercial feasibility is regarded as very low, despite its intense sweetness [77]. The authors report that monellin stability is also limited in carbonated drinks, such as cola. 

Monellin loses its sweetness when heated over 50 °C at an acidic pH. To circumvent such lack of stability, Kim et al. [17] prepared single-chain monellin analogs in which several linkers joined together the two chains. One of these single-chain analogs has been expressed in *E. coli*, has been proven to be as potent a sweetener as the natural product and is stabler under extreme pH and heat conditions.

New insights gained into the structure–activity relation of sweet-tasting protein “protein sectors” can supply meaningful guidelines for their protein engineering, which can considerably speed up improving their properties and promoting the application of sweet-tasting proteins to food and beverages [51].

The potential of sweet proteins is to substitute sugars in food products by acting as good natural and low-calorie sweeteners [78]. Unlike sucrose, they do not trigger the demand for insulin in diabetics. In humans, sweetness can be perceived owing to taste-specific G-protein-coupled heterodimeric receptors T1R2-T1R3. These receptors recognize different synthetic and natural sweeteners, such as monellin and brazzein. The structural modeling of new sweetener proteins will represent a huge step to further advance knowledge and to know their utility as sweeteners to generate low-calorie food [78].

There are also sustainability aspects of using monellin. Cultivation studies have been performed with *Dioscoreophyllum*, except in natural habitats, such as tobacco, to obtain stabile monellin [12]. Therefore, biotransformation studies ought to be performed. A specific form of monellin confers biotransformation flexibility. By way of example, the transformation of monellin via *E. coli* provides a sweet flavor during heating with better pH stability than the original compound [26]. Biotransformation studies with monellin will be necessary to discover sustainable solutions for wide applications to employ its components to manufacture food.

## 4. Safety and Quality Control

A rise in diseases linked with the metabolism of carbohydrates, such as obesity or diabetes, has been linked with dietary changes [73]. According to some studies, increased food consumption has a stronger impact on obesity levels than low energy use. Overeating sucrose, particularly from beverages with added sugar, is one of the key factors to consider when analyzing high food intake. Thus, to promote optimum human health, it is crucial to decrease sucrose intake in diet [79]. The food and beverage industry is now more interested than ever in novel sugar substitutes as a result of this [73]. Even if numerous studies indicate that sweeteners are safe and adequate alternatives to sugar, recent research shows that artificial types of these sweeteners can lead to oxidative stress, metabolic syndrome, nervous system illnesses and changes in the gastrointestinal microbiota [80].

Sweet proteins are a group of sweetening substances that do not share any structural or sequence similarities, despite them all being highly palatable [73] Currently, seven natural sweet-tasting proteins have appeared to date as possible low-calorie sugar alternatives to be used in food and drinks, which include monellin and brazzein [79]. These proteins have been recently studied and have similar sensory features: thaumatin, mabinlin, pentadin, egg white lysozyme, curculin and one sweet-tasting modifying protein, miraculin. Apart from the obvious benefits for avoiding dental cavities or not raising insulin levels, the advantages of protein sweeteners are that they are more nutritious, are safe to consume and that their incredible sweetening power permits only a small amount to be used [18]. Generally speaking, natural peptide sweeteners have fewer negative effects than synthetic types [80]. Nevertheless, the structural and sequence similarities between sweet proteins and allergens suggest that these plant proteins may have allergenic potential. Their ability to cause allergies after intake still has to be conclusively shown [81]. In addition, the ability to create sweet proteins by recombinant technologies opens the door to large-scale manufacturing in heterologous hosts, and their nature allows their sequences to be modified for the best sweetness, among other advantages [18,73].

### 4.1. Brazzein

Brazzein has been used to safely sweeten food by African natives for a long time, which indicates that there are no specific health risks associated with this sweet protein [82,83]. However, before any new food can be consumed by people in the USA, the Food and Drug Administration (FDA) must first approve it. For a new commercial product, the FDA mandates a battery of rigorous testing to guarantee its safety. Research is needed into the similarity of acute toxicity areas to toxins/allergens, plus protein breakdown assays in response to digestive enzyme therapy [75]. For this reason, brazzein’s bioactivity has been tested in vitro and in vivo. Despite exhibiting at least 45% similarity to the antimicrobial agent defensin and antifungal drug drosomycin, brazzein possesses no antibacterial and antifungal activities [82]. Due to the structural resemblance between the sweetener and these defensins, many of which are allergens, concerns have been voiced about the sweetener’s possible allergenicity. This protein is the smallest sweetener of all proteins: it is made up of 54 amino acids, 6473 Da. Brazzein shows a similarity to the knottin motif allergens Art v 1 from mugwort (*Artemisia vulgaris*) and Amb a 4 from ragweed (*Ambrosia artemisifolia*) [81]. Brazzein does, however, display anti-allergic and anti-inflammatory properties during the inhibition of hexosaminidase (IC50 > 15 M) and cyclooxygenase-2 (IC50 = 12.62 M), respectively. There are also potent antioxidant properties to demonstrate DPPH activity (IC50 > 30 M) and ABTS radical scavenging activity (IC50 = 12.55 M). These findings imply that brazzein is a very promising functional sweetener and exhibits anti-inflammatory, anti-allergic and antioxidant effects [82].

In a recent study, 10% sucrose or 3M-brazzein were both dissolved in water for 15 weeks to replicate the development of human obesity in mice. Liquid 3M-brazzein consumption had no effect on adiposity hypertrophy but led to 33.1 ± 0.4 g body weight and 0.90 ± 0.2 mm fat formation, versus 35.9 ± 0.7 g and 1.53 ± 0.067 mm that correspond to a sucrose supplement, respectively. Furthermore, 3M-brazzein did not influence insulin resistance, inflammation or glucose homeostasis maintenance. The results of this study support the notion that drinks with added sugar can play a significant role in the onset of obesity and altered metabolic conditions [79].

Brazzein can be employed as an optimal sweetener for its small size, stability conferred by four disulfide bridges, good solubility and its susceptibility to heat treatment with no denaturation [18]. It can be employed directly in food preparation in its pure form or be expressed in heterologous hosts. Some researchers have identified the areas that contribute to brazzein’s interactions with taste receptors to assist in the identification of those regions that may be altered to obtain the best results [18]. In addition to the aforementioned studies, a thorough physico-chemical study of the recombinant protein, a thorough explanation of the finished product and an exhaustive production process description are additional regulatory criteria. Obtaining regulatory approval should be a very simple process if the chemical brazzein obtains the generally recognized as safe (GRAS) status, but further information of pure proteins is needed, including purification procedure specifics [75]. The FDA must still approve brazzein before it can be sold as a sugar substitute [84]. In February 2022, Sweegen announced the launch of its latest development in sweeteners: Ultratia^TM^ brazzein. With this new sweetener, food and beverage companies can start developing new products that are healthier because they contain less sugar (sweegen.com).

Another peculiar characteristic of using proteins as sweeteners (i.e., brazzein, monellin, egg white lysozyme, curculin/neoculin, pentadine, mabinlin and thaumatin) is their environmental vulnerability, such as pH and temperature changes when they are applied in the food industry. Of these eight proteins, brazzein is the most promising and most appealing sugar substitute in the food industry, because it has the most sugar-like taste and exceptional heat stability.

### 4.2. Monellin

Recently, food and beverage sectors have shown more interest in monellin [85]. Although using monellin as a natural sweetener would be a practical option to substitute refined sugars, currently it is not utilized because it has no legal status with either the European Food Safety Authority (EFSA) or the FDA [83]. Several safety standards must be applied before monellin is approved. Monellin is comparable to other cystatins in structure and sequence terms, but oddly enough, it resembles animal cystatins more than plant cystatins. The allergens with the closest evolutionary ties are animal-derived ones, including cystatin from egg whites and feline D-3 [81]. However, numerous research works reveal that proteins from sweet plants do not cause allergies or hazardous consequences [75]. As far as we are aware, no studies have been conducted nor are currently underway to determine the possible impact of such natural sweeteners on gut flora [86].

In order to extend large-scale berry production to extract sweeteners and to subsequently market them in the food sector, cultivation is inevitable, because *D. cumminisii* berries are uncommon and obtaining huge amounts of them from a wild plant is challenging [83]. Studies about using protein engineering techniques to increase the sweetness level and recombinant production yields of these proteins are still in progress. In addition, figuring out how monellin affects the food matrix into which it is introduced as regards physico-chemical, functional, textural and sensory consequences is a crucial study area to develop new food formulations. Monellin is expected to be manufactured in huge quantities and utilized increasingly more often in the food sector as a natural sweetener in forthcoming years [84].

## 5. Nutritional Profile and Health Impacts

### 5.1. Brazzein

Berlec et al. [42]. discovered brazzein for the first time with the obli fruit of *P. brazzeana* (a west African plant). The compacted structure generated by the four disulfide linkages is responsible for retaining the sweetness profile, even after a 4 h incubation at 353 K. It can be expressed in *E. coli* and transgenic plants [87,88].

Many transgenic cell lines, including those based on *Lactococcus lactis*, yeast, *E. coli*, *Kluyveromyces lactis* and mice, have been reported by studies to synthesize plant-derived brazzein from bacterial and animal cells [42,45,89].

Recombinant brazzein has been produced and purified in former studies by expression in *K. lactis* and *E. coli*. Recombinant brazzein’s sweetness was 1800-fold sweeter than that of sucrose, and even sweeter than the original brazzein, when the crucial residues of its derivatives were modified [18]. Of the mutants, the brazzein with three mutations (H31R/E36D/E41A) was 18-fold sweeter than the wild-type brazzein and was 22500-fold sweeter than sucrose [28,89]. The brazzein generated by *K. lactis* has anti-allergenic, antioxidant and anti-inflammatory properties, which make it desirable for food processing uses [82].

Additionally, plant-mediated biosystems, which include rice, lettuce and maize, are documented [24,25,76]. Yet, employing intact transgenic plants is required for these systems, which restricts their use for field cultivation. Han et al. [90] recently developed a brazzein synthesis platform. To achieve this, they employed carrot cell suspension in a bioreactor. Cell proliferation rapidly increased up to 15 days in the TC12 culture phase and the highest cell division rate was obtained after 6 days. By applying 220 µM H_2_O_2_ and 50 µM ABA, gene expression was 2.5- and 2.8-fold higher than the control, respectively. The resultant transgenic cells were utilized in a variety of air-lift bioreactors, with column bioreactors producing more biomass (238.9 g L^−1^) than balloon and cone bioreactors.

### 5.2. Monellin

Red berries from the West African plant *Dioscoreophyllum cumminsii* Diels contain monellin. Monellin, which Yan et al. purified [45], is used in the food industry as a sweetener and a flavor enhancer. It is around 3000-fold sweeter than sucrose. It displays a series of special benefits over artificial sweeteners, including low calorie content, safety, not introducing artificial metabolites into the body and preserving the amino acid pool balance, and is relatively easy to clone in microorganisms [91]. This protein, unlike single-chain thaumatin, is made up of two polypeptides with 45 and 50 amino acid residues connected by non-covalent interactions. At an acidic pH, it loses its capacity to sweeten above 50 °C. Tyo et al. [92] created single-chain monellin analogs by synthesizing several linkers to bind two chains together to solve the stability problem. When producing one of these single-chain derivatives in *E. coli*, it came over as a potent sweetener and was extremely stable at high pH and temperature values. Additionally, efforts have been made to express monellin in *E. coli*, *Saccharomyces cerevisiae* and *Candida utilis* [64]. Using a T7 phage promoter, Vigues et al. [91] produced a synthetic gene to express monellin in an *E. coli* host. According to the biased codons in *E. coli*, a single-chain monellin gene was produced to maximize the protein’s production. These findings showed that 45% of all soluble proteins are produced as monellin. After purification, it produced 43 mg of protein per gram of dry cell weight.

In a recent study by Cancelliere et al. [93], the authors investigated the metabolic effects of MNEI consumption on a Wistar rat model of high-fat-diet-induced obesity in an effort to shed light on the potential of MNEI as a fructose alternative in beverages in a typical Western diet. They examined the lipid profile, insulin sensitivity and other metabolic syndrome symptoms. Additionally, potential colon damage and systemic inflammation were assessed. Consuming MNEI reversed the metabolic abnormalities brought on by fructose consumption, including insulin resistance, and altered the plasma lipid profile, colon inflammation and the translocation of lipopolysaccharides from the gut lumen into the bloodstream. This study concluded that MNEI might be a good substitute for fructose, especially in cases where concomitant metabolic diseases such as diabetes and/or glucose intolerance are present. 

## 6. Conclusions

The detrimental effects of artificial sweeteners on human health and their negative consequences on the environment are well-established. As reviewed in this article, the naturalness and environment-friendly options offered by brazzein and monellin make them suitable candidates to be considered potential food ingredients in the food industry. Owing to their health benefits, such as anti-inflammatory, anti-allergenic and antioxidant properties, consumers are willing to choose these healthier and natural alternatives.

The method employed to isolate, purify and the further characterize brazzein and monellin will have significant consequences on their functional roles when they are considered alternative sweeteners or ingredients in the food industry. To date, there is some evidence for progress in the commercial application of brazzein. Hopefully, similar progress with monellin will be made, which is subject to food safety authorities’ approval.

In the future, the successful introduction of brazzein and monellin as food ingredients in food industry applications will help to meet consumer demands for healthy, all-natural ingredients.

## Figures and Tables

**Table 1:** Extraction, isolation, purification and characterization methods of the wild-type, synthetic and recombinant brazzein. Wild-type brazzein is referred to as the brazzein extracted from *P. brazzeana*.

Source	Extraction	Isolation	Purification	Characterization	Main Results	Ref.
*P. brazzeana*	0.1 M phosphate buffer at pH 7.0 containing 5% glycerol, 0.1 mM DTT, 20 mL PMSF, 0.1 mM EDTA and 0.5% (*w*/*v*) PVP at 4 °C	Protein precipitation with ammonium sulfate 30% and 85%	Ion-exchange chromatography using a CM-Sepharose CLdB column (gradient: NaCl of 0.1 to 0.4 M in 20 mM sodium citrate at pH 3.6)	SDS- PAGE; ESI-MS; sequence determination by S-Pyridylethylation and S-carboxymethylation of brazzein and peptide fragment separation by RT-HPLC.	Brazzein is a single-chain polypeptide; the molecular weights obtained by SDS-PAGE and ESI-MS were 6.5 KDa and 6.473 KDa, respectively; C-terminal is a tyrosine; 8 cysteines out of 54 residues.	[34]
*Nicotiana tabacum cv.* Xanthi	0.1 M phosphate buffer at pH 7.0 containing 5% glycerol, 0.1 mM DTT, 20 ml PMSF, 0.1 mM EDTA and 0.5% (*w*/*v*) PVP at 4 °C	Protein precipitation with ammonium sulfate 30% and 85%	C_18_ RT-HPLC	^1^H NMR	The secondary structure: 1 α-helix, one short 3_10_ -helix, two strands of antiparallel β-sheet, and probably a third strand near the N-terminal; The core of the brazzein structure is a “cysteine-stabilized alpha-beta” (CSαβ) motif; The tertiary structure stabilized by four disulfide bonds.	[35]
*P. brazzeana*	Buffer solution (40 mM Tris-HCl, 50 mM NaCl, 20 mM EDTA, 55 mM sodium citrate, and 12 mM sodium thiosulfate, pH 6.7)	Ammonium sulfate 30–80% precipitation; heat treatment (80 °C for 2 h)	DEAE-Sepharose anion-exchange chromatography (gradient: 0 to 1.0 M NaCl in 20 mM Tris-HCl at pH 8.0); CM-Sepharose cation-exchange chromatography (gradient: 50 mM sodium acetate buffer with 400 mM NaCl at pH 4.0)	SDS-PAGE; RP-HPLC; CD; N-terminal amino acid sequencing; ESI-MS/MS.	The expressed brazzein presents a molar mass of 6.5 KDa; Elution time on RT-HPLC is identical to the brazzein expressed from *K. lactis*; The secondary structure: 9.9% of α-helices and 19.7% of β-sheets.	[36]
*P. brazzeana*	0.1 M phosphate buffer at pH 7.0 containing 5% glycerol, 0.1 mM DTT, 20 mL PMSF, 0.1 mM EDTA and 0.5% (*w*/*v*) PVP at 4 °C	Protein precipitation with ammonium sulfate 30% and 85%	Ion-exchange chromatography using a CM-Sepharose CLdB column (gradient: NaCl of 0.1 to 0.4 M in 20 mM sodium citrate at pH 3.6)	^1^H NMR (pH 5.2; 22 °C)	Folding is due to the ‘cysteine-stabilized alpha-beta’ (CSαβ) motif stabilizing the α-helix by two disulfide bonds with the nearest β-strand; Total of four disulfide bonds responsible for protein folding and its sweetness.	[37]
*P. brazzeana*	0.1 M phosphate buffer at pH 7.0 containing 5% glycerol, 0.1 mM DTT, 20 mL PMSF, 0.1 mM EDTA and 0.5% (*w*/*v*) PVP at 4 °C	Protein precipitation with ammonium sulfate 30% and 85%	HPLC (mobile phase composed of 0.05% TFA (A) and acetonitrile with 0.05% TFA gradient: 10% B to 18% B obtained in 55 min, 18% B to 25% B in 65 min and 25% B to 10% B in 75 min; flow rate of 10 mL/min)	X-ray Crystallography (pH 4; 293 K) to 1.8 Ă resolution	The first brazzein crystal to 1.8 Ă resolution is reported.	[38]
*Escherichia coli (E. coli)*	0.1 M phosphate buffer at pH 7.0 containing 5% glycerol, 0.1 mM DTT, 20 mL PMSF, 0.1 mM EDTA and 0.5% (*w*/*v*) PVP at 4 °C	Protein precipitation with ammonium sulfate 30% and 85%	HPLC (mobile phase composed of 0.05% TFA (A) and acetonitrile (B) with 0.05% TFA gradient: 10% B to 18% B obtained in 55 min, 18% B to 25% B in 65 min and 25% B to 10% B in 75 min; flow rate of 10 mL/min)	X-ray Crystallography (pH 4; 293 K) to 1.8 Ă resolution	The crystal structure is composed of one short α -helix and three β-strands which form a triple-stranded antiparallel β–sheet; it also contains an additional α-helix that is absent in the brazzein solution structure; In solution, brazzein exists as a monomer; In crystal, brazzein forms a homodimer stabilized by six hydrogen bonds.	[39]
*E. coli*	-	-	Nickel-affinity chromatography (mobile phase: 0–500 mM Imidazol in PBS) Cation-exchange chromatography (SP-Sepharose column; gradient: 30–1000 mM NaCl)	X-ray Crystallography (pH 4–4.5; 291 K)	Structures of the recombinant brazzein exhibit two α -helices and three β-strands linked by four disulfide bonds with a significantly altered electrostatic distribution on the surface.	[40]
*E. coli*	Tris–HCl buffer 50 mM (pH 8.0, with 2 mM EDTA)	-	CM-cellulose ion-exchange chromatography (mobile phase: 50 mM Tris-HCl with 0.6 M NaCl, pH 7.6); RT-HPLC	NMR (25 °C)	The recombinant protein adopts a cysteine-stabilized αβ (CSαβ) fold stabilized by 17 inter-strand α-helical hydrogen bonds and four disulfide bridges, that together contribute to the marked heat (100 °C) and cold (216 °C) stability of brazzein within a pH range of 2.5–11.0.	[41]
*E. coli*	Tris-HCl buffer 50 mM (pH 8.0, with 2 mM EDTA)	-	CM-cellulose ion-exchange chromatography (mobile phase: 50 mM Tris-HCl with 0.6 M NaCl, pH 7.6); RT-HPLC	RT-HPLC; NMR (pH 5.2; 37 °C).	Compared to the wild-type protein, the mutated brazzein displays an extended β-structure due to the terminal β-sheets and increased dynamics.	[42]
*Kluyveromyces lactis*	-	-	Nickel-affinity chromatography	CD (pH 7.6, 25 °C); Intrinsic fluorescence; ANS fluorescence.	Recombinant proteins (E9K and E9G) presented a molar mass of 6.5 KDa; The secondary structure of E9K brazzein is stabler than E9K and the wild-type brazzein; Brazzein has 6 tyrosine residues at positions 8, 11, 24, 39, 51 and 54, and a phenylalanine residue at position 38; The tertiary structure of the recombinant proteins is more compact than the wild-type brazzein; The local tertiary structure around tyrosine residues in the wild-type brazzein and E9G brazzein is more exposed to a polar environment;	[43]
*Pichia pastoris*	-	-	CM-Sepharose cation-exchange chromatography (gradient: 100 to 1000 mM NaCl IN 50 mM sodium acetate, at pH 4.0; flow rate of 1 mL/min)	SDS-PAGE; RT-HPLC (mobile phase: 0.1% TFA and 70% acetonitrile with 0.1% TFA); CD (25 °C).	104 mg/L can be obtained from the recombinant brazzein; The molar mass of recombinant protein is 6.5 KDa with an elution time in RT-HPLC of 9 ± 0.5 min; Compared to the wild-type brazzein, no significant alterations in the secondary structure of recombinant brazzein are observed by CD analysis.	[44]
*Bacillus licheniformis*	-	-	Cation-exchange chromatography (SP-Sepharose column; gradient: 0 to 1 M NaCl in 50 mM sodium acetate buffer pH 4 in 50 min; flow rate of 1 mL/min)	SDS-PAGE; ESI-MS; NMR.	Recombinant proteins are correctly folded;	[45]
*E. coli*	-	-	Cation-exchange chromatography (Q-Sepharose column; gradient: 300 to 1000 mM NaCl in 20 mM Tris buffer; flow rate of 1 mL/min)	SDS-PAGE; LC-MS/MS; CD (25 °C).	85% purity; formation of disulfide bonds is confirmed by LC-MS/MS; The secondary structure of the recombinant protein is similar to the wild-type brazzein.	[46]
*E. coli*	-	-	Nickel-affinity chromatography	SDS-PAGE; LC-MS/MS; MALDI-TOF.	Formation of disulfide bonds is confirmed by LC-MS/MS; The secondary structure of the recombinant protein is similar to the wild-type brazzein	[46]
*Lactococcus lactis*			Talon-affinity chromatography; RT-HPLC.	Edman degradation; SDS-PAGE.	The primary structure of the recombinant brazzein is similar to the wild-type brazzein.	[47]
			Talon-affinity chromatography; RT-HPLC.	Edman degradation; SDS-PAGE.	The primary structure of the recombinant brazzein is similar to the wild-type brazzein.	[47]

DTT: dithiothreitol; PMSF: phenyhnethylsulfonyl fluoride; EDTA: ethylenediamine tetra acetic acid; PVP: polyvinylpolypyrrolidone; CM: carboxymethyl; SDS-PAGE: sodium dodecyl sulfate-polyacrylamide gel electrophoresis; RP-HPLC: reverse-phase high-performance liquid chromatography; ESI-MS: electrospray ionization mass spectrometry; NMR: Nuclear Magnetic Resonance; TFA: trifluoroacetic acid; SP: sulfopropyl; ANS: 8-Anilino naphthalene 1-sulfunate; DEAE: diethylaminoethyl.

**Table 2:** Extraction, isolation, purification and characterization methods of the wild-type, synthetic and recombinant monellin. Wild-type monellin is referred to as the monellin extracted from *Dioscoreophyllum cumminsii* Diels. * MNEI monellin is a single-chain monellin composed of the two chains of naturally occurring monellin linked by a Gly-Phe dipeptide.

Source	Extraction	Isolation	Purification	Characterization	Main Results	Ref.
*D. cumminsii*	-	Salt precipitation	Ion-exchange chromatography	SDS- PAGE; Gel filtration; Fluorescence spectroscopy.	The molecular weight of monellin obtained by SDS-PAGE and gel filtration is 10.5 KDa and 10.0 KDa, respectively. The primary structure has 91 amino acids with single residues of tryptophan, methionine, and cysteine	[54]
*D. cumminsii*	-	Salt precipitation; Gel filtration on Sephadex G-50 (mobile phase: 1% aqueous acetic acid).	Ion-exchange chromatography; Affinity chromatography (mobile phase: 6 M guanidine. HCl, 0.1 M sodium phosphate buffer, pH 7.4, containing 0.2 M dithiothreitol).	SDS-PAGE; Edman degradation.	Monellin is composed of two chains of similar length linked by non-covalent bonds. However, the subunits, devoid of sweetness, are not identical. By abolishing the thiol group, monellin loses its sweet taste	[58]
Standard	-	-	Ion-exchange chromatography using a Sephadex-CM 25-gel (mobile phase: 100 mM NaCl, 10 mM phosphate buffer); RT-HPLC (gradient: 30% methanol, 0.1% TFA to 70% methanol, 0.1% TFA).	N-terminal amino acid sequencing; ESI-MS/MS; Size-exclusion chromatography; NMR; CD	The secondary structure: in the native state, the chain A of monellin consists of β-structure, and chain B contains both α and β-structures; Addition of 50% ethanol or TFE denatures the protein.	[60]
*Synthesized*	-	-	RT-HPLC; HIC.	HPLC; ESI-MS; Quantitative amino acid analysis.	Monellin contains five Aspartate residues and nine Lysine residues; Asp^87^ h plays an important role in monellin sweetness.	[61]
MNEI monellin * expressed in *E. coli*	-	-	Ion-exchange chromatography using a HiPrep26/60 Sephacryl 100 column (mobile phase: sodium acetate and sodium chloride); Gel filtration in a G-75 column (elution: 150 mM ammonium bicarbonate).	X-ray Crystallography (resolution of 1.15 Ă)	The crystal contains a single MNEI protein in the asymmetric unit and lacks the dimer interface observed in all the previous crystal structures of monellin and its single-chain derivatives; Four stably bound negative ions are also located and can be related to potential electrostatic interactions with the surface of the sweet taste receptor;	[56]
*MNEI and muted MNEI monellin expression in E. coli*	-	-	Ion-exchange chromatography; Size-exclusion chromatography.	SDS-PAGE; CD.	Mutated protein eluted at high salt concentrations (200 mM NaCl) relative to MNEI monellin (100–150 mM NaCl). CD spectra of the mutated monellin presents two minimums at 201 and 213 nm; At pH 2.5–6.8, the β-sheet and α-helix content exhibit minor changes, which corroborates the folding stability of the mutated monellin	[59]
*E. coli*	-	-	Ion exchange chromatography; Size-exclusion chromatography.	ESI-MS; Hydrogen exchange-mass spectrometry.	Monellin purity exceeds 95%. Chain A and B molar masses are 5.382 KDa and 5.965 KDa, respectively; Double-chain monellin (dcMN) unfolds in a barrier-limited manner in which chain B undergoes non cooperative exchange and chain A cooperatively.	[62]
*E. coli*	-	Incubation of the cell extract at 60 °C for 10 min and at pH 4 for 1 h at 4 °C	Ion-exchange chromatography using a Sephadex CM-50 column (gradient: 0 to 0.4 M NaCl); SDS-PAGE.	-	Recombinant monellin yield of 43 mg/g of dry cell wt. Purity confirmed by SDS-PAGE.	[63]
*Candida utilis*	-	-	Ion exchange chromatography using a CM-Sepharose column (gradient: 0–0.4 M NaCl);	SDS-PAGE	Molecular mass of single-chain protein is 10.0 KDa. In total soluble protein, 5% is monellin.	[64]
*E. coli*	-	-	Ion-exchange chromatography using CM-cellulose and DEAE-cellulose columns due to the nature of the different mutants proteins; RT-HPLC using a Resource RPC column (mobile phase: eluent A composed of 10% acetonitrile/water with 0.1% TFA, and eluent B of 90% acetonitrile/water with 0.1% TFA; gradient: 20–50% B).	SDS-PAGE; Amino acid analysis followed by hydrolysis; MALDI-TOF-MS; NMR-HSQC spectroscopy; Fluorescence emission spectroscopy; CD.	The intermolecular and intramolecular Coulombic interactions are involved in the stabilization of recombinant monellin and in the reconstitution of the wild-type monellin. Charge interactions may significantly modulate the folding of monellin and its binding to the sweet receptors by modifying association rates.	[65]
*E. coli*	-	-	Nickel-affinity chromatography; RT-HPLC (mobile phase: acetonitrile 5%; flow rate of 1 mL/min).	SDS-PAGE	H-Monellin shows an identical fold and a typical β-sheet-rich structure. The molar mass of H-monellin is 16.0 KDa, with 14.0 KDa for the MNEI monellin.	[66]

TFE: trifluoroethanol; HIC: hydrophobic interaction chromatography.
